# Favourable outcomes for high-risk diffuse large B-cell lymphoma (IPI 3–5) treated with front-line R-CODOX-M/R-IVAC chemotherapy: results of a phase 2 UK NCRI trial^✰^

**DOI:** 10.1016/j.annonc.2020.05.016

**Published:** 2020-09

**Authors:** A.K. McMillan, E.H. Phillips, A.A. Kirkwood, S. Barrans, C. Burton, S. Rule, R. Patmore, R. Pettengell, K.M. Ardeshna, A. Lawrie, S. Montoto, S. Paneesha, L. Clifton-Hadley, D.C. Linch

**Affiliations:** 1 Haematology Department, Nottingham University Hospitals NHS Trust, Nottingham, UK; 2 Cancer Research UK and UCL Cancer Trials Centre, UCL Cancer Institute, University College London, London, UK; 3 Division of Cancer Sciences, University of Manchester and The Christie Hospital NHS Trust, Manchester, UK; 4 HMDS, St James' University Hospital, Leeds, UK; 5 Plymouth University Medical School, Plymouth, UK; 6 Haematology Department, Castle Hill Hospital, Hull, UK; 7 Clinical Sciences, St George's University of London, London, UK; 8 Haematology Department, University College Hospital London, London, UK; 9 Centre for Haemato-oncology, Barts Health NHS Trust, London, UK; 10 Haematology Department, Heart of England NHS Trust, Birmingham, UK; 11 UCL Cancer Institute, University College London, UK

**Keywords:** chemotherapy, diffuse large B-cell lymphoma (DLBCL), double-hit, high-grade B-cell lymphoma, R-CODOX-M

## Abstract

**Background:**

Outcomes for patients with high-risk diffuse large B-cell lymphoma (DLBCL) treated with R-CHOP chemotherapy are suboptimal but, to date, no alternative regimen has been shown to improve survival rates. This phase 2 trial aimed to assess the efficacy of a Burkitt-like approach for high-risk DLBCL using the dose-intense R-CODOX-M/R-IVAC regimen.

**Patients and methods:**

Eligible patients were aged 18–65 years with stage II–IV untreated DLBCL and an International Prognostic Index (IPI) score of 3–5. Patients received alternating cycles of CODOX-M (cyclophosphamide, vincristine, doxorubicin and high-dose methotrexate) alternating with IVAC chemotherapy (ifosfamide, etoposide and high-dose cytarabine) plus eight doses of rituximab. Response was assessed by computed tomography after completing all four cycles of chemotherapy. The primary end point was 2-year progression-free survival (PFS).

**Results:**

A total of 111 eligible patients were registered; median age was 50 years, IPI score was 3 (60.4%) or 4/5 (39.6%), 54% had a performance status ≥2 and 9% had central nervous system involvement. A total of 85 patients (76.6%) completed all four cycles of chemotherapy. There were five treatment-related deaths (4.3%), all in patients with performance status of 3 and aged >50 years. Two-year PFS for the whole cohort was 67.9% [90% confidence interval (CI) 59.9–74.6] and 2-year overall survival was 76.0% (90% CI 68.5–82.0). The ability to tolerate and complete treatment was lower in patients with performance status ≥2 who were aged >50 years, where 2-year PFS was 43.5% (90% CI 27.9–58.0).

**Conclusions:**

This trial demonstrates that R-CODOX-M/R-IVAC is a feasible and effective regimen for the treatment of younger and/or fit patients with high-risk DLBCL. These encouraging survival rates demonstrate that this regimen warrants further investigation against standard of care.

**Trial Registration:**

ClinicalTrials.gov (NCT00974792) and EudraCT (2005-003479-19).

## Highlights

• R-CODOX-M/R-IVAC is an effective regimen for treatment of high-risk DLBCL and high-grade B-cell lymphoma (IPI score 3–5).
• Treatment was well tolerated in patients aged <50 years, or aged 50–65 with performance status 0 or 1.
• The 2-year PFS was 67.9% (90% CI: 59.9–74.6) for the whole cohort.
• This regimen warrants further evaluation against standard of care in high-risk DLBCL.

## Introduction

The addition of rituximab to standard CHOP chemotherapy [cyclophosphamide, doxorubicin, vincristine and prednisolone (R-CHOP)] has improved survival rates for diffuse large B-cell lymphoma (DLBCL) and other forms of high-grade B-cell lymphoma (HGBL) by 10%–15%.^1^, ^2^, ^3^ However, a third of DLBCL patients still progress after R-CHOP, and outcomes for these patients are extremely poor.^4^, ^5^, ^6^ The greatest unmet need is for patients with high-risk disease; R-CHOP failure rates for patients with an International Prognostic Index (IPI) score of 3–5 approach 50%.^7^, ^8^, ^9^, ^10^

There have been extensive, largely unsuccessful attempts to improve on standard 21-day R-CHOP chemotherapy for untreated DLBCL. Increasing the dose density of R-CHOP has not been shown to improve outcomes,^11^ nor has consolidation with high-dose therapy-autologous stem cell transplantation.^12^, ^13^, ^14^ Randomised studies have not shown clear evidence of benefit for incorporation of novel agents for most patients.^15^, ^16^, ^17^, ^18^, ^19^ Several attempts to incorporate additional chemotherapeutic agents have similarly failed to improve outcomes.^20^^,^^21^

There is some evidence, however, that treatment intensification can improve survival. The phase 3 GELA LNH03-2B trial demonstrated an overall survival (OS) advantage for treatment with R-ACVBP (rituximab, doxorubicin, cyclophosphamide, vindesine, bleomycin and prednisolone) plus consolidation chemotherapy over R-CHOP in patients with an age-adjusted IPI score (aaIPI) of 1, albeit with unexpectedly poor outcomes in the R-CHOP arm.^22^ Favourable outcomes have been achieved with the same regimen for high-risk patients (aaIPI 2–3), although it remains unclear whether there is a benefit over R-CHOP in this group.^23^^,^^24^ A number of population-based and non-randomised studies have suggested that combining etoposide with R-CHOP can improve outcomes for high-risk patients, although randomised studies in the rituximab era are lacking.^25^^,^^26^

A different approach is widely used in Burkitt lymphoma (BL), using rapid cycling of dose-dense chemotherapy, combining hyperfractionated alkylating agents with multiple central nervous system (CNS)–penetrating agents.^27^ One such example is the Magrath regimen, consisting of alternating cycles of CODOX-M (cyclophosphamide, vincristine, doxorubicin and high-dose methotrexate) and IVAC chemotherapy (ifosfamide, etoposide and cytarabine),^28^ which, with the addition of rituximab, can achieve survival rates in BL approaching 80%, even in patients with multiple high-risk features.^29^^,^^30^ The LY10 study demonstrated that the same treatment regimen, without rituximab, was both feasible and effective in highly proliferative DLBCL and HGBL.^28^ Two-year OS was 59% with CODOX-M/IVAC in patients with a proliferation rate >95%. The aim of this UK National Cancer Research Institute (NCRI) trial was to build on these encouraging results and assess the efficacy of CODOX-M and IVAC, together with rituximab, for the treatment of high-risk DLBCL.

## Methods

This trial was designed as two parallel single-arm trials to treat both DLBCL and BL patients with the same regimen under the same protocol, with crossover between arms according to central pathology review. Outcomes for DLBCL patients are reported here; outcomes for BL patients will be reported separately.

### Eligibility

Patients were eligible for this phase 2 trial if aged 18–65 years with stage II–IV newly diagnosed DLBCL (or any morphological variant according to World Health Organisation (WHO) Classification of Lymphoid Neoplasms)^31^^,^^32^ and an IPI score of ≥3. Prior corticosteroid treatment was permitted but no other chemotherapy or radiotherapy. Performance status (PS) was permissive, but patients must have been deemed able to tolerate the intensive regimen with adequate renal, liver and cardiac function. A protocol amendment allowed inclusion of HIV-positive patients with PS ≤2 and baseline CD4 count ≥100 cells/mm^3^, with no history of opportunistic infection.

Diagnostic tissue was centrally reviewed by the Leeds Haematological Malignancy Diagnostic Service, UK. Cell of origin was assessed *post hoc* according to the Hans algorithm.^33^ FISH studies for *MYC,* *BCL2* and *BCL6* translocations were not mandated but were routinely performed in patients recruited in later stages of the trial. Disease staging was with contrast-enhanced computed tomography (CT) of the neck to pelvis, bone marrow biopsy and cerebrospinal fluid examination. All patients provided informed consent prior to study entry.

### Study treatment and assessments

Patients received two cycles of CODOX-M alternating with two cycles of IVAC, plus eight doses of rituximab (375 mg/m^2^; Table 1). Subsequent treatment cycles were commenced as soon as neutrophils were >1 × 10^9^/l and platelets >75 × 10^9^/l. Tumour lysis prophylaxis with allopurinol or rasburicase was commenced prior to study treatment. All patients received supportive care with pegylated granulocyte–colony stimulating factor, aciclovir and *Pneumocystis jirovecii* prophylaxis.

**Table 1:** R-CODOX-M and R-IVAC regimen

R-CODOX-M regimen			IVAC regimen		
Rituximab	375 mg/m^2^ i.v.	Days 1 and 11	Rituximab	375 mg/m^2^ i.v.	Day 1
Cyclophosphamide	800 mg/m^2^ i.v. 200 mg/m^2^ i.v.	Day 1 Day 2-5	Etoposide	60 mg/m^2^	Days 1–5
Vincristine	1.5 mg/m^2^ i.v. (max 2 mg)	Days 1 and 8	Ifosfamide	1500 mg/m^2^	Days 1–5
Doxorubicin	40 mg/m^2^ i.v.	Day 1	Mesna	1200 mg	Days 1–5
Cytarabine	70 mg i.t.	Days 2 and 4 Day 6^b^ if CNS disease	Cytarabine	2000 mg/m^2^ i.v. 12 h	Days 1 and 2
Methotrexate	3000 mg/m^2^ i.v. over 24 h^d^	Day 10	Methotrexate	12 mg i.t.	Day 5
Leucovorin	From 36 h after methotrexate until clearance, starting at a dose of 15 mg/m^2^ i.v. 6 h		Pegfilgrastim	6 mg s.c.	Day 7
Pegfilgrastim	6 mg s.c.	Day 13	Cytarabine	70 mg i.t.	Days 7 and 9, if CNS disease^b^
Methotrexate	12 mg i.t.	Day 15 Day 17^b^ if CNS disease	Rituximab^c^	375 mg/m^2^ i.v.	Days 21 and 42

CNS, central nervous system; CODOX-M, cyclophosphamide, vincristine, doxorubicin and high-dose methotrexate; i.t., intrathecal; i.v., intravenous; IVAC, ifosfamide, etoposide and high-dose cytarabine; R-CODOX-M, rituximab + R-CODOX-M; R-IVAC, rituximab + IVAC; s.c., subcutaneous.

^a^ Estimated timeline based on median cycle length.

b First cycle of R-CODOX-M and R-IVAC only, for patients with evidence of CNS disease.

c After fourth cycle of chemotherapy (second IVAC cycle) only.

d 300 mg administered over 1 h followed by 2700 mg over 23 h.

Response was assessed by contrast-enhanced CT according to standard criteria,^34^ 1 month after completion of chemotherapy. Use of positron emission tomography (PET) was encouraged but was not routinely available in the UK at the time of study design. Radiotherapy consolidation was permitted at investigators' discretion to initial sites of disease bulk, intraparenchymal CNS disease and sites of residual positron emission tomography-positive disease. Adverse events were assessed according to the Common Terminology Criteria for Adverse Events version 3.0.

### End points and statistical methods

The primary end point was progression-free survival (PFS) at 2 years. Secondary end points included complete response (CR) rate and toxicity. At the time of study design, PFS for DLBCL patients with IPI 3–5 after standard therapy was estimated to be 40%–50%, based on historical data and assuming a 10%–15% improvement in the rituximab era. A PFS rate of ≥65% was deemed to warrant further investigation but a rate of ≤45% would be of no interest. Using a Fleming design, it was calculated that a sample size of ∼95 patients would have 90% power to detect a 15% difference at 5% (one-sided) significance. The trial was designed to treat both BL and DLBCL patients under one protocol and was terminated once the total target sample size of 150 patients was met, irrespective of numbers of DLBCL patients recruited. PFS was calculated as the time from registration until progression or death. Patients who were alive and progression-free were censored at the date last seen. Competing risks survival analysis was used to calculate the risk of CNS relapse, with death and systemic-only relapse treated as competing events. All analyses were performed using Stata version 15.1 (StataCorp, College Station, TX).

## Results

### Patient characteristics

**Figure 1:**
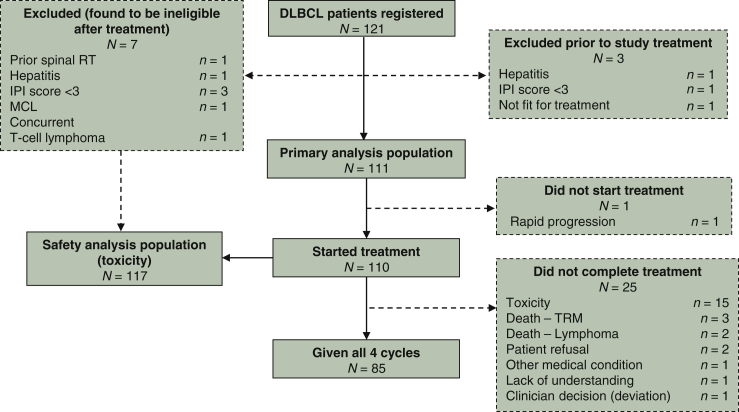
Consort diagram. DLBCL, diffuse large B-cell lymphoma; IPI, International Prognostic Index; MCL, mantle cell lymphoma; RT, radiotherapy; TRM, treatment-related mortality.

Between May 2008 and April 2013, 121 patients were registered with DLBCL at 32 UK centres. Three patients were deemed ineligible prior to treatment and have been excluded from all analyses (Figure 1). An additional seven patients were found not to meet eligibility criteria after starting treatment and have been included in toxicity analyses only. One eligible patient did not commence treatment because of rapid disease progression and was included in survival, but not toxicity, analyses.

The primary analysis included 111 DLBCL patients. Baseline clinical and pathological characteristics are shown in Table 2. Central pathology review was performed in 86 patients (77.5%); pathology reports issued by haematopathologists were reviewed for all other patients to confirm DLBCL. FISH studies were performed equally in germinal centre (GC) B-cell and non-GC DLBCL; seven patients (12.3% of those assessed) had double-hit cytogenetics.

**Table 2:** Baseline characteristics

Baseline characteristic	*N* = 111
Demographics	
Age (years), median (range)	50 (18–65)
Sex, *n* (%)	
Female	45 (40.5)
Male	66 (59.5)
Prognostic factors, *n* (%)	
Age	
≤60	98 (88.3)
>60	13 (11.7)
WHO performance status	
0	23 (20.7)
1	28 (25.2)
2	38 (34.2)
3	22 (19.8)
Stage	
III	7 (6.3)
IV	104 (93.7)
More than 1 extra nodal site	
No	23 (20.7)
Yes	88 (79.3)
LDH above upper limit of normal	
No	5 (4.5)
Yes	106 (95.5)
IPI score, *n* (%)	
3	67 (60.4)
4	43 (38.7)
5	1 (0.9)
Other baseline demographics, *n* (%)	
Age-adjusted IPI score	
1	1 (0.9)
2	54 (48.6)
3	56 (50.5)
B symptoms	
Absent	34 (30.6)
Present	77 (69.4)
CNS disease at registration	
Yes	10 (9.0)
No	101 (91.0)
HIV status	
Negative	108 (99.1)
Positive	1 (0.9)
Unknown	2
Disease bulk ≥10 cm	
Present	38 (39.6)
Absent	58 (60.4)
Incomplete information	15
LDH ≥3× ULN, *n* (%)	
No	60 (56.1)
Yes	47 (43.9)
Unknown	4
Pathology classification (post hoc review), *n* (%)	
Cell of origin	
GCB	54 (54.0)
Non-GCB	46 (46.0)
Unknown	11
Double-hit	
No	50 (87.7)
Yes	7 (12.3)
Unknown	54

CNS, central nervous system; GCB, germinal centre B-cell; HIV, human immunodeficiency virus; IPI, International Prognostic Index; LDH, lactate dehydrogenase; ULN, upper limit of normal; WHO, World Health Organisation.

### Study treatment

Eighty-five patients (77.3%) completed all four cycles of chemotherapy. The median interval between the start of consecutive treatment cycles was 27 days between cycles 1 and 2 (range 18–45), 23 days between cycles 2 and 3 (range 16–53) and 30 days between cycles 3 and 4 (range 21–83). The interval was ≥35 days for 16.5% (44/266) of treatment cycles.

Twenty-five patients (22.7%) stopped treatment early, after receiving one (*n* = 16), two (*n* = 3) or three (*n* = 6) cycles of chemotherapy. The main reason for early discontinuation was toxicity (*n* = 18; Figure 1). Patients were less likely to complete chemotherapy if aged ≥50 years (62.5%, compared with 92.7% <50 years; *P* < 0.001), or if PS = 3 (54.6%, compared with 83.2% for PS 0–2; *P* = 0.004).

Radiotherapy consolidation was given to 13 of 85 (15.3%) patients. Only one of 20 (5%) patients with baseline tumour bulk ≥7.5 cm was irradiated after achieving CR. Two patients received allogeneic stem cell transplant off-trial in CR, one of whom died of transplant-related complications.

### Toxicity

Grade 3–5 adverse events are detailed in Table 3. As expected, haematological toxicity was high with this dose-intense regimen, with 88% grade 4 neutropoenia and 61.1% grade 4 thrombocytopoenia. Grade ≥3 nonhaematological toxicity occurred in 88.9%, the most frequent of which were infections (70.9%), mucositis (31.6%) and febrile neutropoenia (17.9%). Intracranial haemorrhage occurred in six patients (5.1%), all during the first cycle and in patients without CNS disease. One patient (0.9%) developed secondary acute myeloid leukaemia. One patient who had received prior radiotherapy for spinal lymphoma (thus should have been ineligible, although it was only reported after completing study treatment) developed paraparesis secondary to radiotherapy-induced spinal necrosis, emphasising the need for caution with intensive CNS-directed therapy after CNS irradiation.

**Table 3:** Grade 3–5 adverse events

CTCAE system organ class/event	Worst grade	
	Grade 3–4	Grade 5
	*N* = 117	
	*n* (%)	*n* (%)
**Blood and bone marrow**	**115 (98.3)**	
Anaemia	32 (27.4)	
Leukopenia	14 (12.0)	
Neutropaenia	112 (95.7)	
Thrombocytopaenia	110 (94.0)	
**Cardiac**	**12 (10.3)**	
Cardiac NOS	10 (8.5)	
Hypotension	2 (1.7)	
**Constitutional**	**26 (22.2)**	
Fatigue	7 (6.0)	
Fever	20 (17.1)	
**Dermatology and skin: rash**	**2 (1.7)**	
**Gastrointestinal**	**56 (47.9)**	
Anorexia	12 (10.3)	
Diarrhoea	10 (8.5)	
Mucositis	37 (31.6)	
Nausea	14 (12.0)	
Perforated small bowel	2 (1.7)	
Vomiting	7 (6.0)	
**Haemorrhage**	**9 (7.7)**	**2 (1.7)**
CNS	5 (4.3)	1 (0.1)
Gastrointestinal	4 (3.4)	1 (0.1)
**Infection**	**80 (68.4)**	**3 (2.6)**
Febrile neutropoenia	21 (17.9)	
Infection	72 (61.5)	3 (2.6)
Sepsis	3 (2.6)	
**Laboratory/Metabolism**	**20 (17.1)**	
Hypokalaemia	7 (6.0)	
Abnormal transaminases or bilirubin	8 (6.8)	
**Neurology**	**16 (13.7)**	
Mood alterations	2 (1.7)	
Neurological NOS	11 (9.4)	
**Pain**	**18 (15.4)**	
Gastrointestinal	3 (2.6)	
Musculoskeletal	5 (4.3)	
Headache	7 (6.0)	
Pain NOS	4 (3.4)	
Chest	3 (2.6)	
**Pulmonary/Upper respiratory**	**11 (9.4)**	
Dyspnoea	4 (3.4)	
Pleural effusion	3 (2.6)	
**Syndromes**	**3 (2.6)**	
Tumour lysis	2 (1.7)	
**Vascular**	**3 (2.6)**	
DVT/thrombosis	3 (2.6)	
**Non-haematological**	**99 (84.6)**	**5 (4.3)**
**Any CTCAE grades 3+**	**112 (95.7)**	**5 (4.4)**

Data listed by organ class according to the Common Toxicity Criteria for Adverse Events version 3. Individual grade ≥3 events are only listed if occurring in ≥2 patients.

CNS, central nervous system; CTCAE, Common Toxicity Criteria for Adverse Events (version 3.0); DVT, deep vein thrombosis; NOS, not otherwise specified.

Treatment-related mortality (TRM) was 4.3%, with five deaths, due to neutropoenic sepsis (*n* = 3) and haemorrhage (*n* = 2; intracranial and gastrointestinal). These patients were all aged >50 years with a PS of 3 at registration.

### Outcomes

For eligible patients that commenced R-CODOX-M (*N* = 110), overall response rate by CT was 74.5%: 52 patients (47.3%) achieved CR/unconfirmed CR (CRu) and 30 (27.3%) achieved partial response (PR). Seven patients (8.2%) had stable or progressive disease and 20 patients (18.2%) did not undergo response assessment because of early treatment termination or death (Figure 1).

**Figure 2:**
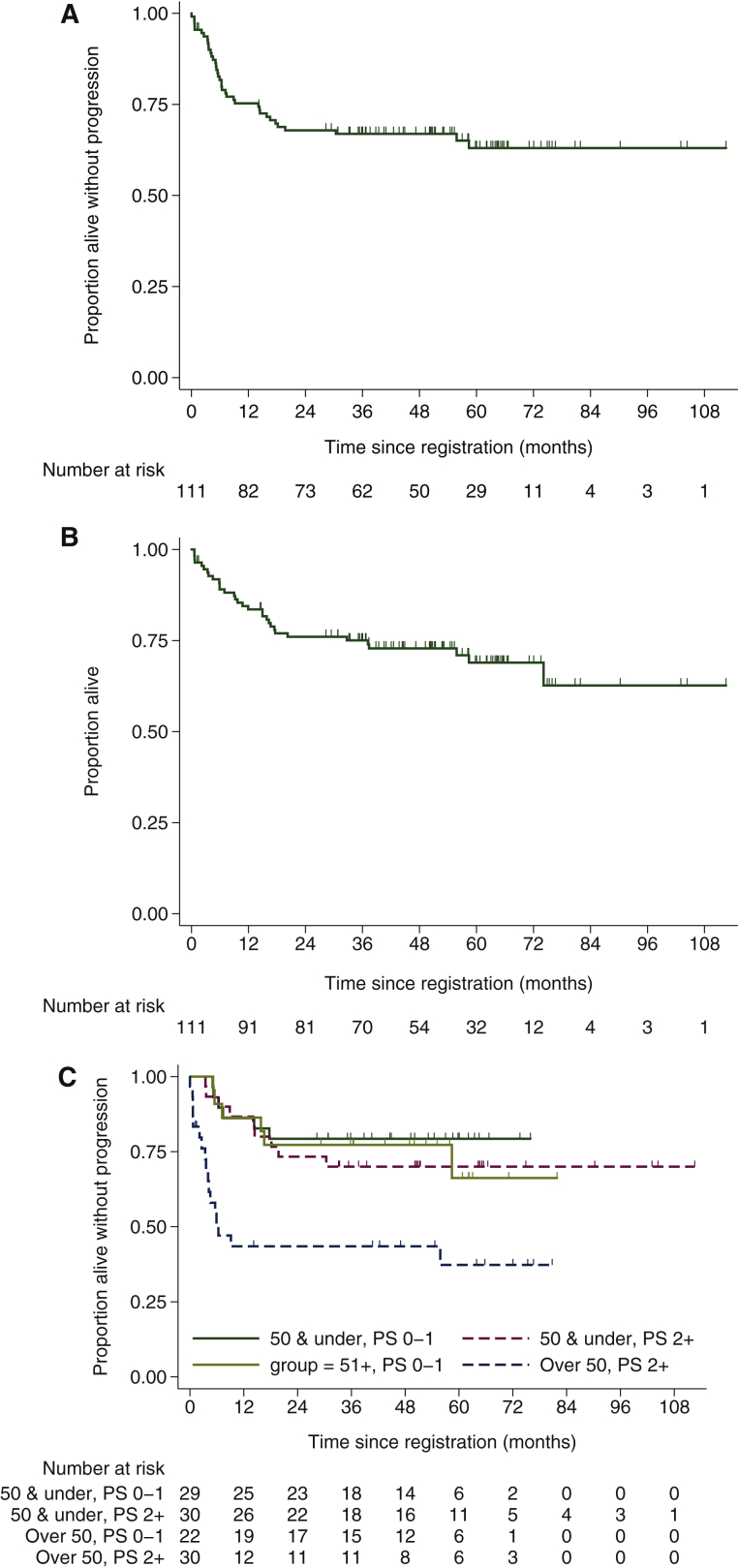
Kaplan–Meier curves for (A) progression-free survival and (B) overall survival. (C) Progression-free survival according to age and performance status (PS).

With a median follow-up of 54.6 months for the whole cohort (*N* = 111), 30 patients have relapsed or died of lymphoma and eight died without progression (38 PFS events). The 2- and 4-year PFS rates were 67.9% [90% confidence interval (CI) 59.9–74.6] and 66.9% (90% CI 58.9–73.7), respectively (Figure 2A). The 2- and 4-year OS rates were 76.0% (90% CI 68.5–82.0) and 72.8% (90% CI 64.9–79.2), respectively (Figure 2B). There have been 32 deaths in total, due to lymphoma (*n* = 22), TRM (*n* = 5), toxicity of further treatment (*n* = 3), pneumonia (*n* = 1) and other malignancy (T-cell lymphoma; *n* = 1).

A *post hoc* analysis assessing the effect of age and PS highlighted worse outcomes for patients with PS ≥2 who were aged >50 years (Figure 2C), which was largely driven by excess TRM in this group (supplementary Table S1, available at *Annals of Oncology* online). There was no clear difference in outcomes for those with IPI score 3 compared with those with IPI score 4 or 5 (supplementary Figure S1A, available at *Annals of Oncology* online). There was also no overt difference in outcomes for DLBCL diagnoses made by external versus central pathology review, or between patients with a GC B-cell phenotype and non-GC disease (supplementary Figure S1B and C, available at *Annals of Oncology* online), albeit according to the Hans algorithm, which is an imperfect predictor of cell of origin. Although numbers are small, outcomes for patients with double-hit lymphoma were not overtly different from other DLBCL patients in whom FISH excluded double-hit disease (supplementary Figure 1D, available at *Annals of Oncology* online).

#### Central nervous system-directed therapy

For patients with CNS involvement at registration (*n* = 10), 2-year PFS was 70% (32.9%–89.2%), without any patient receiving radiotherapy or high-dose therapy and stem cell transplant in CR. There was one isolated CNS progression; the other two patients had either refractory disease or concurrent systemic progression.

For patients without evidence of CNS involvement (*N* = 101), CNS relapse risk according to the CNS-IPI^35^ was evaluable for 90 (89.1%; supplementary Table S2, available at *Annals of Oncology* online). There were no CNS relapses in intermediate-risk patients (*n* = 38). There were two CNS relapses among 58 high-risk patients, both without evidence of systemic disease, giving a 2-year CNS relapse rate of 3.6% (95% CI 0.9–13.8).

#### Outcomes for relapsed/refractory disease

Twenty-six patients experienced disease progression after R-CODOX-M/R-IVAC, nine of whom relapsed from CR/CRu, and two patients received further treatment for inadequate response (PR and stable disease). Of these, 20 patients (71.4%) received intensive salvage chemotherapy, five (17.9%) were palliated and further treatment history is unknown in three (10.7%). Seven patients with relapsed/refractory disease (25.0%) are alive, with a minimum of 29 months' follow-up post-progression.

## Discussion

This trial demonstrates that treatment with R-CODOX-M/R-IVAC is both feasible and effective in a high-risk group of DLBCL/HGBL patients. The primary end point was met, with a 2-year PFS rate of 67.9% (90% CI 59.9–74.6), exceeding the 65% target and comfortably excluding the lower limit of 45%.

Our results appear to compare favourably with contemporary outcomes with R-CHOP in high-risk DLBCL, notwithstanding the inherent limitations of making comparisons between studies. We report a 4-year PFS rate of 66.9% (90% CI 58.9–73.7), despite inclusion of patients with CNS involvement, in whom R-CHOP is ineffective, and patients with PS >2, who are excluded from many prospective trials.^11^ In the UK NCRI R-CHOP 14-21 trial,^11^ 5-year PFS for high-risk DLBCL patients (IPI 3–5), aged ≤60 years with PS 0–2 was 54.4%.^10^ In other large cohorts of patients treated with R-CHOP or similar regimens, PFS rates at 4–5 years for patients with IPI score 3 and 4 or 5 were 54%–59% and 41%–56%, respectively.^8^^,^^9^ Outcomes in this trial were similar to those reported with other intensive treatment regimens.^24^^,^^25^ We observed a 4-year OS rate of 72.8%, compared with 78% for high-risk patients (aaIPI 2–3) treated with the R-ACVBP regimen, noting that CNS disease was excluded in the latter and fewer patients (58%) had IPI score 3–5.^24^

The main limitation of this trial is the lack of a randomised comparator, particularly in light of the failure of other apparently promising regimens to translate into a survival benefit over R-CHOP in phase 3 trials.^16^^,^^17^^,^^19^^,^^21^ Our findings must therefore be interpreted with caution and randomised studies are clearly required if this regimen is to be brought forwards into standard care for high-risk DLBCL. However, conducting a large phase 3 trial in this relatively small subset of DLBCL patients with widespread disease and poor PS, many of whom require urgent chemotherapy, will be challenging.

Toxicity with R-CODOX-M/R-IVAC was greater than expected with R-CHOP, particularly with respect to haematological toxicity, infection and mucositis, but was manageable for most patients. TRM was 4.3%, which is comparable to TRM rates with other intensive treatment strategies^12^^,^^22^^,^^24^ but higher than the 1%–2% TRM seen in most R-CHOP trials.^11^^,^^21^ All treatment-related deaths occurred in patients aged >50 years with PS 3. These deaths and higher rates of early treatment discontinuation highlight the need for caution in using this regimen for older patients (>50 years) with PS ≥2. A corticosteroid prephase may improve PS and allow for better assessment of suitability for intensive treatment. Use of published dose-adjusted CODOX-M/IVAC protocols for older/less-fit patients may also improve tolerability,^28^^,^^36^ although alternative regimens may be warranted for such patients. The resource burden with R-CODOX-M/R-IVAC is higher than with R-CHOP, usually requiring inpatient administration, but the duration of treatment (14 weeks) is shorter than with R-CHOP-21 (18 weeks) and other intensive regimens, such as R-ACVBP (26 weeks).^22^

One benefit of R-CODOX-M/R-IVAC is the ability to deliver multiple CNS-directed agents, particularly to those with concurrent CNS involvement or at highest risk of CNS relapse. The favourable outcomes for those with CNS involvement in this trial demonstrate the feasibility of this approach for secondary CNS lymphoma, although patient numbers are small, and our findings require exploration in larger cohorts. It is also noteworthy that none of these patients received high-dose therapy and stem cell transplant or radiotherapy consolidation in CR. There were also fewer CNS relapses than anticipated in this trial, with observed 2-year CNS progression rates of 0% and 3.6% for patients with intermediate- and high-risk CNS IPI scores, respectively, compared with 3.4%–3.9% and 10.2%–12.0%, respectively, in R-CHOP-treated patients.^35^

R-CODOX-M/R-IVAC has already been used to treat specific high-risk groups of HGBL, particularly those with ‘double-hit’ lymphoma, based on retrospective evidence of efficacy.^37^^,^^38^ Only 51.4% of patients underwent FISH studies in this trial, which was conceived prior to the widespread use of FISH to identify double-hit patients. Therefore, our findings cannot be extrapolated to recently categorised HGBL populations in the 2016 WHO Classification of Lymphoid Neoplasms.^39^ Where FISH studies were performed, the frequency of double-hit cytogenetics in our high-risk DLBCL cohort (12%) was not appreciably higher than the reported incidence in wider DLBCL cohorts (5%–10%). There was no clear difference in outcomes for the few patients with double-hit lymphoma in this trial. This demonstrates that adverse risk is multifactorial and double-hit lymphoma accounts for only a small proportion of ‘high-risk’ HGBL. Our findings suggest that R-CODOX-M/R-IVAC may have broader applicability across high-risk patients with both HGBL and DLBCL.

One argument against the use of intensive upfront treatment is the limited availability of treatment options in the event of relapse; R-IVAC contains several agents that are typically used in salvage regimens. However, outcomes after DLBCL progression are generally poor, with PFS rates following intensive salvage therapy of only 20%–30% across studies.^4^, ^5^, ^6^ Even with chimeric antigen receptor T-cell therapy, relatively short-term PFS rates for the selected patients included in pivotal trials remain below 40% on a per-protocol basis, and intention-to-treat analyses are lacking.^40^^,^^41^ In this trial, 25% of all patients with relapsed/refractory disease are alive and in ongoing remission, in keeping with other studies and emphasising the importance of effective front-line treatment.

In summary, this phase 2 trial demonstrates that R-CODOX-M/R-IVAC is an effective regimen for the treatment of high-risk DLBCL, with promising survival rates. Toxicity with this intensive regimen was manageable for most patients, although it was less well tolerated in patients aged >50 years with impaired PS (≥2). Our findings indicate that R-CODOX-M/R-IVAC warrants being explored further in comparative studies against standard R-CHOP chemotherapy.
