# Supplementary material for: Favourable outcomes for high-risk diffuse large B-cell lymphoma (IPI 3–5) treated with front-line R-CODOX-M/R-IVAC chemotherapy: results of a phase 2 UK NCRI trial
Source: Ann Oncol. 2020 Sep;31(9):1251–9. doi: 10.1016/j.annonc.2020.05.016 (PMC7487775; doi:10.1016/j.annonc.2020.05.016)

**Supplementary Table 1: Outcomes by age and performance status**

|  | | **≤50 yrs, PS 0-1** | | **≤50 yrs, PS 2+** | **>50 yrs, PS 0-1** | **>50 yrs, PS 2+** |
| --- | --- | --- | --- | --- | --- | --- |
|  | | | **N=29** | **N=30** | **N=22** | **N=30** |
|  | | |  |  |  |  |
| **PFS event** | | | **6 (20.7)** | **9 (30.8)** | **6 (27.3)** | **17 (56.7)** |
| **Death** | | | **5 (17.2)** | **7 (23.3)** | **5 (22.7)** | **15 (50.0)** |
| **Causes of death** | | |  |  |  |  |
|  | **Non-Hodgkin's Lymphoma** | | *4* | *7* | *3* | *8* |
|  | **Other - post progression ^1^** | | *1* | *0* | *0* | *1* |
|  | **Secondary Malignancy** | | *0* | *0* | *1* | *0* |
|  | **Other ^2^** | | *0* | *0* | *1* | *1* |
|  | **Treatment-related toxicity** | | *0* | *0* | *0* | *5* |

PFS: progression-free survival; PS: performance status

^1^ deaths were both due to infection, following further treatment

^2^ deaths were both due to allogeneic stem cell transplant complications (1 in complete remission, 1 for secondary acute myeloid leukaemia)

**Supplementary Table 2: Risk Factors for CNS relapse according to CNS-IPI in patients without baseline CNS disease**

| **IPI** | |  |
| --- | --- | --- |
|  | 3 | 59 (58.4) |
|  | 4 | 41 (40.6) |
|  | 5 | 1 (1.0) |
| **Renal or adrenal involvement at baseline, N (%)** | |  |
|  | No | 63 (70.0) |
|  | Yes | 27 (30.0) |
|  | Unknown | 11 |
| **CNS-IPI, N (%)** | |  |
|  | 3 | 38 (42.2) |
|  | 4 | 41 (45.6) |
|  | 5 | 10 (11.1) |
|  | 6 | 1 (1.1) |
|  | Unknown | 11 |
| **CNS-IPI (group), N (%)** | |  |
|  | Intermediate risk (2-3) | 38 (39.6) |
|  | High risk (4-6) | 58 (60.4) |
|  | Unknown | 5 |

CNS: central nervous system; IPI: international prognostic index

**Supplementary Figure 1: Kaplan-Meier curves for progression-free survival according to A) IPI, B) central pathology review, C) cell-of-origin (Hans algorithm, excluding double-hit patients) and D) double-hit status.**

**A**


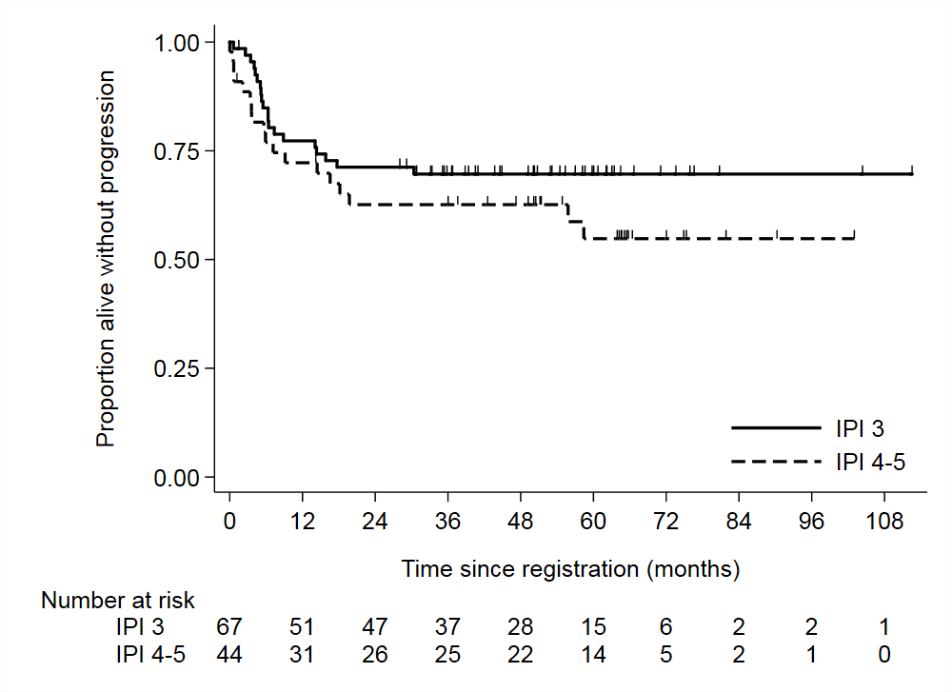


**B**

**
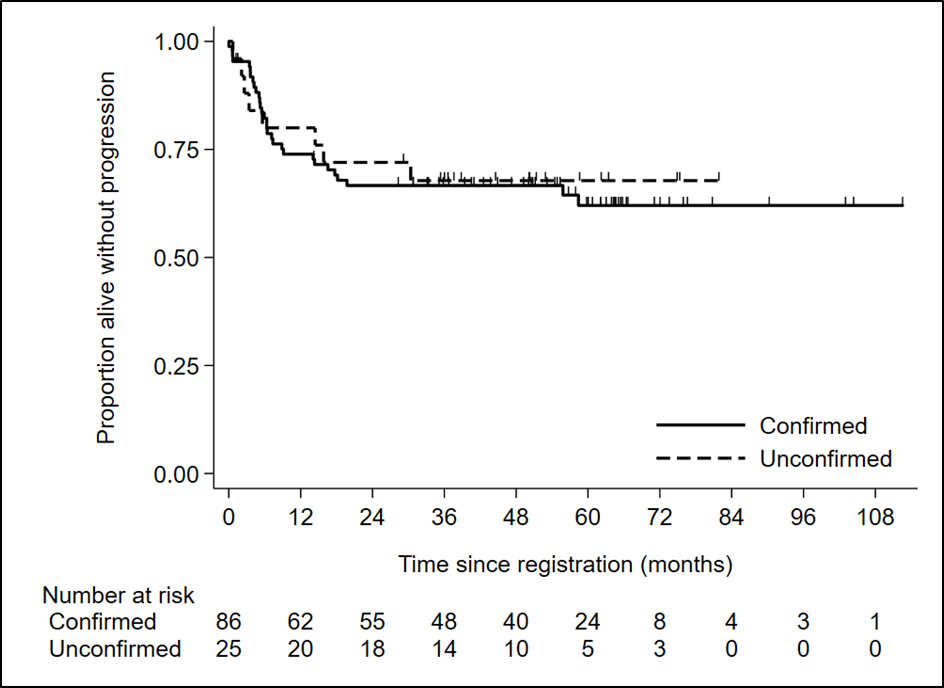
**

**C**


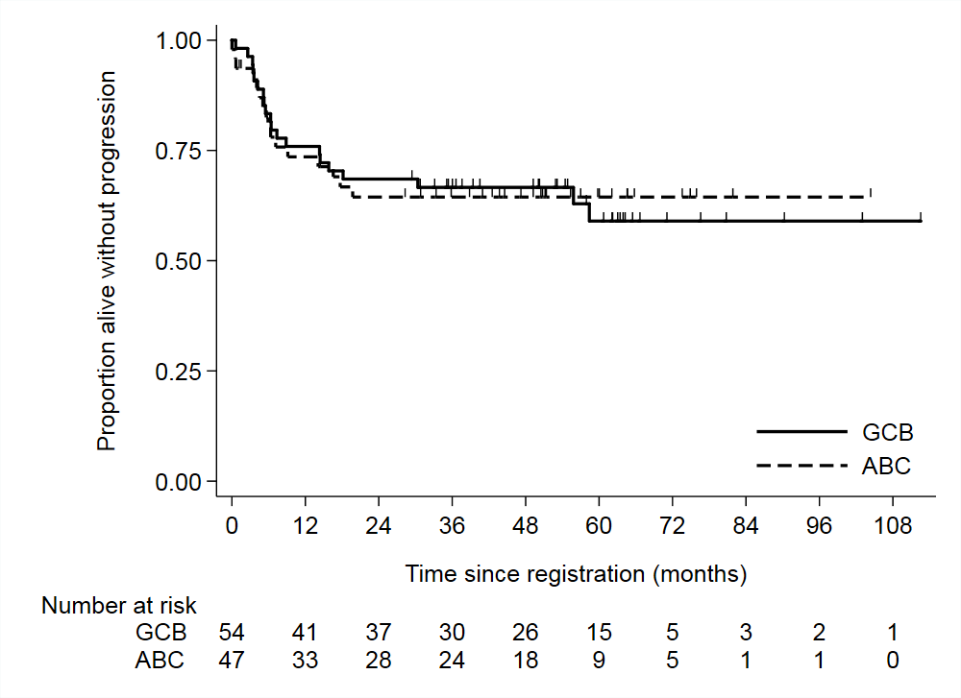


**D**


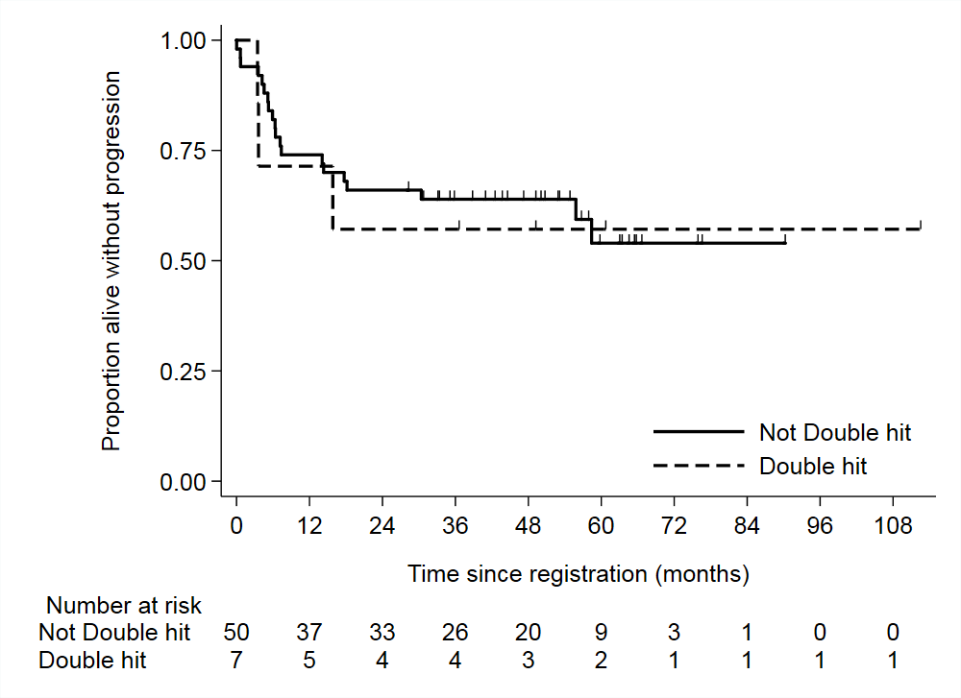

Supplement: Supplementary Material [file mmc1.docx]
